# Cognitive performance of children with spinal muscular atrophy: A systematic review

**DOI:** 10.1590/1980-57642018dn13-040011

**Published:** 2019

**Authors:** Graziela Jorge Polido, Mariana Mangini Vaz de Miranda, Nelson Carvas, Rodrigo de Holanda Mendonça, Fátima Aparecida Caromano, Umbertina Conti Reed, Edmar Zanoteli, Mariana Callil Voos

**Affiliations:** 1 Programa de Pós-Graduação em Ciências da Reabilitação, Faculdade de Medicina, Universidade de São Paulo, SP, Brasil.; 2 Departamento de Neurologia, Departamento de Neurologia Clínica, Universidade Federal de São Paulo, SP, Brasil.; 3 Programa de Pós-Graduação em Ciências da Saúde do Instituto de Assistência Médica ao Servidor Público Estadual de São Paulo, SP, Brasil.

**Keywords:** spinal muscular atrophy, cognition, cognitive impairment, systematic review, atrofia muscular espinhal, cognição, revisão sistemática

## Abstract

Spinal muscular atrophy (SMA) is genetic and progressive, caused by large bi-allelic deletions in the SMN1 gene, or the association of a large deletion and a null variant.

**Objective:**

To evaluate the evidence about cognitive outcomes in spinal muscular atrophy (SMA).

**Methods:**

Searches on the PUBMED/Medline, Web of Knowledge and Scielo databases retrieved 26 studies (1989 to 2019, descriptors “spinal muscular atrophy” and “cognition”). Nine studies were selected according to the eligibility criteria: (1) cognition tested in individuals with SMA; (2) written in English or Spanish. The Risk of Bias in Non-Randomized Studies of Interventions was used to describe design, bias, participants, evaluation protocol and main findings. This study was registered on the International prospective register of systematic reviews (PROSPERO).

**Results:**

Three studies described normal cognition. In another three studies, cognitive outcomes were above average. Cognitive impairment was found in three studies. Poor cognitive performance was more frequently reported in studies that were recent, included children with SMA type I and that employed visual/auditory attention and executive function tests. Protocols and cognitive domains varied, precluding metanalysis.

**Conclusion:**

The severity of motor impairment may be related to cognitive outcomes: studies that included a higher number/percentage of children with SMA type I found cognitive impairment. The establishment of gold-standard protocols is necessary. Further studies should compare the cognitive outcomes of subjects with SMA types I to IV.

## RESUMO

A atrofia muscular espinhal (SMA) é genética e progressiva, causada por grandes deleções bi-alélicas no gene SMN1, ou pela associação de uma grande deleção e uma variante nula.

**Objetivo:**

Avaliar as evidências sobre o desempenho cognitivo na atrofia muscular espinhal (AME).

**Métodos:**

Pesquisas nas bases de dados PUBMED/ Medline, Web of Knowledge e Scielo localizaram 26 estudos (1989 a 2019, descritores “atrofia muscular espinhal” e “cognição”). Nove estudos foram selecionados de acordo com os critérios de elegibilidade: (1) testaram a cognição em pessoas com AME; (2) escritos em inglês/espanhol. A avaliação do risco de viés em estudos com intervenções não-randomizadas foi utilizada para descrever o desenho experimental, viés, amostra, protocolo de avaliação e principais achados. Este estudo foi aprovado no Registro Internacional Prospectivo de Revisões Sistemáticas (PROSPERO).

**Resultados:**

Em três estudos, foi registrado que a cognição estava preservada. Em três estudos, o desempenho cognitivo estava acima da média. O comprometimento cognitivo foi encontrado em três estudos. Desempenho cognitivo pobre foi mais frequentemente relatado em estudos recentes, estudos que incluíram crianças com AME tipo I e estudos que incluíram atenção visual/auditiva e testes de função executiva. Protocolos e domínios cognitivos variaram muito, portanto não foi possível a realização de metanálise.

**Conclusão:**

A gravidade do comprometimento motor pode estar relacionada ao desempenho cognitivo: estudos que incluíram maior número/porcentagem de crianças com AME tipo I encontraram alterações no desempenho cognitivo. O estabelecimento de protocolos padrão-ouro é necessário. Novos estudos devem comparar o desempenho cognitivo de pessoas com AME tipos I a IV, ou seja, com diferenças no prognóstico e no desempenho motor.

Spinal muscular atrophy (SMA) is genetic and progressive, caused by large bi-allelic deletions in the SMN1 gene, or the association of a large deletion and a null variant.^1^ SMA leads to the degeneration of the anterior horn motor neurons and the motor nuclei of some cranial nerves.^1^^,^ 2 Only in rare cases have two single nucleotides changes been reported to be causative of the disease. The most common form is caused by mutations in the survival motor neuron 1 (*SMN1*) gene (5q11.2) and SMA is transmitted in an autosomal recessive fashion.^1^^,^ 2

SMA classification is based on the age of onset and the maximal function attained.^1^^,^ 2 Children classified as type 0 exhibit almost complete paralysis and have very short survival time.^3^ Children who do not acquire sitting are classified as type I (non-sitter), with onset in the first semester of life (Werdnig-Hoffmann disease). Type II (sitter) usually begins before age of 18 months and includes children who can sit without support. Type III (walker) is considered a juvenile form of SMA, with standing and gait acquisition. Type IV begins in adulthood. In all types of SMA, sensory function is normal.^1^^,^ 2 ^,^ 4 The SMN2 gene can produce a small amount of the functional protein expressed by the SMN1 gene. This gene performs some phenotypic rescue in SMA, and the phenotype severity is determined by the number of SMN2 copies harbored by the patient. SMA type 1 can harbor 1 or 2 copies of SMN2, and patients with fewer copies are more severely impaired.^3^

For many years, researchers focused on motor and respiratory outcomes of children with SMA and cognitive performance was not investigated. In 2006, D´Angelo and Bresolin^5^ stated that the cognitive development of affected children had not received much attention and that most studies relied on anecdotal clinical reports and did not follow gold-standard neuropsychology guidelines. More than ten years on, cognitive outcomes of children with SMA still need to be reviewed.

SMA (mainly types I and II) is associated with severe weakness, which impacts hand coordination and speech acquisition.^1^^,^ 2 Restricted verbal and sensorimotor interaction can cause cognitive impairment.^6^ In a previous study, children with SMA type I and age- and sex-matched controls were evaluated with an eye-tracking device on pair-matching tasks. A relationship between the pair-matching performance and social function, scored by the Pediatric Evaluation Disability Inventory, was found.^6^ However, cognitive outcomes in SMA are contradictory in the literature. For instance, the study by von Gontard (2002)^7^ reported normal intelligence coefficients in children, and even above normal cognitive performance in adolescents with SMA types I-III.^7^

The present study aimed to describe and discuss the evidence on cognitive outcomes in children with SMA. Difficulties exploring objects and the environmental, together with poor oral communication, may impair cognitive development. We performed a systematic review to assess the cognitive performance of children with SMA and possible differences in cognitive performance among types I, II and III.

## METHODS

### Search strategy and selection criteria

This study was registered on the International prospective register of systematic reviews (PROSPERO) and followed the checklist of the Preferred Reporting Items for Systematic Reviews and Meta-Analyzes (PRISMA).^8^ A search over the last 30 years (1989 to 2019) was carried out and included studies from PUBMED/Medline, Web of Knowledge and Scielo (Scientific Electronic Library Online) databases. The searches were performed from 11 July 2015 to 07 April 2019.

The search with the descriptors “cognition” and “spinal muscular atrophy” resulted in the retrieval of 36 studies on PUBMED/Medline and 17 studies on Web of Science/Web of Knowledge. The search with the same descriptors retrieved no studies on the Scielo database. Of the 53 studies identified, 10 were eliminated because they were duplicates. There were no restrictions on the types of study design.

The titles and abstracts of 43 studies were independently screened by two investigators (GJP and MCV). Twenty-three studies were excluded because they did not evaluate people with SMA (there was a consensus between GJP and MCV on all excluded studies).

The same investigators then evaluated and discussed the inclusion of each of the twenty selected studies. They adopted as inclusion criteria: quantitative assessment of cognitive skills; studies written in English or Spanish. All studies met the inclusion criteria.

As exclusion criteria, the investigators considered: review studies (n=5 excluded; two about cognition in several types of neuromuscular diseases and three about locomotion in SMA); letter to the editor (n=1); studies with no cognitive assessment (n=5). Therefore, nine studies were selected for this systematic review. 

### Data extraction and quality assessment

Given the nine selected studies were non-randomized, they were analyzed by the Risk of Bias in Non-Randomized Studies of Interventions (ROBINS-I).^9^ ROBINS-I is a tool for evaluating the risk of bias of non-randomized studies, whose results may be biased by sampling and interventions. ROBINS-I evaluated the risk of bias of the research question and the outcome and results. It also examined how the confounders and co-interventions were addressed and the risk of bias judgements.^9^

We classified bias with ROBINS-I guidelines and described the design, bias, participants, evaluation protocol, and main findings of the selected studies (Table 1). The eligibility and bias of each study were independently evaluated by two investigators (GJP and MCV). All items scored differently by the examiners were discussed and rescored with a consensual classification.

**Table 1:** Risk of Bias in Non-Randomized Studies - of Interventions (ROBINS-I).9

Authors/Year/Title/ Study design/Overall bias	Participants	Evaluation protocol	Main findings	Risk of bias
**Billard C, Gillet P, Signoret JL, Uicaut E, Bertrand P, Fardeau M, et al., 1992^10^** *Cognitive functions in Duchenne muscular dystrophy: a reappraisal and comparison with spinal muscular atrophy* Cross-sectional **Moderate risk of bias**	Duchenne muscular dystrophy (n=24): 12 to 16 years (mean 14 years); spinal muscular atrophy (n=17) types II and III): 12 to 16 years (mean 14 years). No control group.	Assessment was made by a single-blinded evaluator. Three cognitive areas were assessed: **Language** (MacCarthy intelligence scale for children, North syntactic screening test, Alouette test for reading age and the verbal scale of the Wechsler intelligence scale: information, similarities, arithmetic and vocabulary); **Visuospatial** (the performance scale of the Wechsler intelligence scale: picture completion, picture arrangement, block design and object assembly); **Attention-memory** (selective auditory response and *Batterie d'Efficience Mnésique*: global verbal and visual memory).	Normal performance of children with spinal muscular atrophy. Children with SMA had higher scores on Wechsler intelligence scale (normal level) than children with Duchenne muscular dystrophy (below normal). Children with SMA had higher scores on the North syntactic screening test and on the global verbal and visual memory. Only 61% of the participants with Duchenne muscular dystrophy had normal reading age compared to 100% of participants with SMA.	Muscle strength/function and cranial nerve functions were not assessed and may have influenced results. **Bias due to confounding.** No control group: spinal muscular atrophy was compared to Duchenne muscular dystrophy. Children with spinal muscular atrophy type I were not included. Education and social class were not controlled. **Bias in selection of participants.** Sometimes the examiner helped the patient with hand manipulation. Tests with motor components and/or time limits were used. **Bias in measurement of outcomes.**
**Authors/Year/Title/Study design/Overall bias**	**Participants**	**Evaluation protocol**	**Main findings**	**Risk of bias**
**Billard C, Gillet P, Barthez M-A, Hommet C, Bertrand P, 1998^11^** *Reading ability and processing in Duchenne muscular dystrophy and spinal muscular atrophy* Cross-sectional **Moderate risk of bias**	Duchenne muscular dystrophy (n=21): 8 to 13 years (mean 11 years); spinal muscular atrophy I and II (n=11): 6 boys, 8 to 13 years (mean 11 years). Control group (n=42): 22 boys, 6 to 14 years (mean 9 years).	Wechsler intelligence scale: information, similarities, vocabulary and arithmetic **(verbal tasks);** picture completion, picture arrangement, object assembly and block design **(non-verbal tasks):** only children with spinal muscular atrophy and Duchenne muscular dystrophy were tested. **Language:** *Batterie d'evaluation du langage;* (word repetition, picture naming); *Test de Vocabulaire Actif et Passif* (word comprehension); North syntactic screening test. **Reading ability:** *La pipe et le rat;* reading aloud test.	Normal performance of children with spinal muscular atrophy. Children with spinal muscular atrophy and with Duchenne muscular dystrophy did not differ in Wechsler intelligence scale total scores. Children with Duchenne muscular dystrophy had lower scores in similarities and arithmetic (verbal tasks). Children with spinal muscular atrophy had significantly higher reading skills than children with Duchenne muscular dystrophy.	Muscle strength/function and cranial nerve functions were not assessed and may have influenced results. **Bias due to confounding.** Children with spinal muscular atrophy who were able to read but had severe motor impairment were excluded from the protocol. Education and social class were not controlled. **Bias in selection of participants.** Wechsler intelligence scale was not applied in the control group. **Bias due to missing data.** Tests with motor components and/or time limits were used. **Bias in measurement of outcomes.**
**Authors/Year/Title/Study design/Overall bias**	**Participants**	**Evaluation protocol**	**Main findings**	**Risk of bias**
Rivière,Lécuyer, 2002^12^ *Spatial cognition in young children with spinal muscular atrophy* Cross-sectional **Low risk of bias**	Spinal muscular atrophy type II (n=12, 7 boys, 2 years). 12 healthy children (1 year and 6 months), 12 healthy children (2 years), 12 healthy children (2 years and 6 months), 12 healthy children (3 years).	A **spatial location memory test** was used. A toy was hidden in one of three cups aligned horizontally on a table. The tabletop was rotated 180 degrees and the child participated in three trials. The task was to find the hidden toy.	Normal performance of children with spinal muscular atrophy. There was no difference between groups. All groups showed normal spatial location performance. Locomotor impairment does not appear to be a risk factor for slowing down the development of spatial perception.	This study evaluated only children with one type of spinal muscular atrophy (type II). Diagnosis was not genetically confirmed. **Bias in selection of participants.** This study explored a very specific developmental phase. Each trial ended when the child had searched for the hidden toy (correct versus incorrect location). Therefore, only Chi-square tests were possible. **Bias in measurement of outcomes.**
**von Gontard A, Zerres K, Backes M, Laufersweiler-Plass C, Wendland C, Melchers P, et al., 2002^7^** *Intelligence and cognitive function in children and adolescents with spinal muscular atrophy* Cross-sectional **Low risk of bias**	96 children and adolescents with Spinal muscular atrophy: 18 with type I, 58 with type II and 20 with type III (6 to 18 years); 45 non-affected siblings: mean age 11 years; 59 healthy children without affected siblings: mean age 11 years.	Raven colored and standard progressive matrices and Kaufman assessment battery for children(6 to 11 years): **spatial perception, memory, executive function, arithmetic, reading comprehension.** Wechsler intelligence scale (12 to 18 years): **reading comprehension, arithmetic, executive function, vocabulary, memory.**	Normal/above normal performance of children with spinal muscular atrophy. Children and adolescents with spinal muscular atrophy have normal intelligence. Adolescents with spinal muscular atrophy showed higher intelligence than controls, probably due to environmentally mediated factors (strategies to compensate for their physical handicap). There were no differences between the performances of types I, II and III.	Of a sample of 261 patients, 128 died (mostly type I) and six were excluded due to language difficulties. Some patients classified as type I were borderline between types I and II. Only 83 patients underwent genetic testing. **Bias in selection of participants.**
**Authors/Year/Title/Study design/Overall bias**	**Participants**	**Evaluation protocol**	**Main findings**	**Risk of bias**
Rivière,Lécuyer, 2003^13^ *The C-not-B error: a comparative study* Cross-sectional Moderate risk of bias	14 children with spinal muscular type II, aged 1 to 3 years (mean 2 years, 6 boys) and 14 controls (7 boys).	A **spatial location memory** test involving invisible displacements of an object was used. A toy was hidden under one of three cloths. The child had to find the hidden toy. The misplacement of the hidden toy is known as the C-not-B error.	Normal/above normal performance of children with spinal muscular atrophy. The performance of children with spinal muscular atrophy was better than the performance of the control group. Inhibitory mechanisms present in children with spinal muscular atrophy are not as well-developed in healthy children, who tend to show more impulsive responses.	Reaching and grasping are critical for this task. Therefore, muscle strength may have influenced movement inhibition. **Bias due to confounding.** This study evaluated only children with one type of spinal muscular atrophy (type II). Diagnosis was not genetically confirmed. **Bias in selection of participants.** Each trial ended when the child had searched for the hidden toy (correct versus incorrect location). Therefore, only Chi-square tests were possible. **Bias in measurement of outcomes.**
**Authors/Year/Title/ Study design/Overall bias**	**Participants**	**Evaluation protocol**	**Main findings**	**Risk of bias**
**Chung, Wong, Ip, 2004^15^** *Spinal muscular atrophy: survival pattern and functional status* Cohort study **Moderate risk of bias**	Children and adults with spinal muscular atrophy (n=83), aged 1 to 40 years (22: type I, 9 boys; 26: type II, 9 boys; 16 type IIIa, 9 boys; 19 type IIIb, 7 boys)	Parents/caregivers were interviewed for the scoring of the functional independence measure for children. The questionnaire consisted of three domains: selfcare, mobility and cognition. The cognition domain evaluated comprehension, expression, social interaction, problem solving, and memory and the maximum score was 35. This domain aimed to assess the real-life performance in daily activities, which required **executive function.**	The performance of children with spinal muscular atrophy was below normal. The percentage of individuals who had achieved functional cognitive independence were SMA II: 60%; SMA IIIa: 78%; spinal muscular atrophy IIIb: 90%. The cognition scores of participants with SMA types III and II were higher than the scores of participants with type I.	Education and social class were not controlled. **Bias due to confounding.** There was no control group. Some participants were not genetically tested for spinal muscular atrophy. **Bias in selection of participants.** Intervention discontinuation was likely to be related to prognostic factors: higher mortality among more severe cases (probably type I). **Bias due to missing data.** Only one domain of the functional independence measure for children evaluated cognition and no other scales were used. **Bias in measurement of outcomes.**
Rivière, Lécuyer, Hickmann, 2009^14^ *Early locomotion and the development of spatial language: evidence from young children with motor impairments* Cross-sectional Moderate risk of bias	12 children with spinal muscular atrophy type II (2 to 3 years, mean age 3 years) and 12 controls.	The acquisition of **language** (understanding and production of specifically **spatial terms**) was evaluated in a series of 22 pictures. The understanding and production of the words in, on, under, above, in front of, behind, near, far, in the upper region, in the lower region were tested (in French).	Normal/above normal performance of children with spinal muscular atrophy. There was no difference between the two groups on the comprehension task. In the production task, the SMA group had better performance than controls when producing the words for the relations in front of and behind.	Only one type of SMA was evaluated. Participants were not genetically tested for SMA. **Bias in selection of participants.** This study explored a very specific developmental phase and some tasks were too difficult for both groups. **Bias in measurement of outcomes**. Chi-square tests compared the percentage of correct answers but no data about variability were presented. **Bias in selection of the reported result.**
**Authors/Year/Title/Study design/Overall bias**	**Participants**	**Evaluation protocol**	**Main findings**	**Risk of bias**
**Barja S, Muñoz C, Cancino N, Núnez A, Ubilla M, Sylleros R, et al., 2013^16^** *Estimulación audiovisual en niños con limitación grave de la motricidade: ?mejora su calidad de vida?* Case series **Serious risk of bias**	3 children with spinal muscular atrophy type I; 2 with neuropathy; 3 with myopathy. All children were classified as V in the Gross Motor Function Classification System	Audiovisual stimuli were used for evaluation/training (4 weeks, twice a day). **Attention** was assessed by Tobii P10 with the software Catch-me (gaze viewer, eye-tracking device). Animation figures were based on the country's flag and other images while the sounds were based on farm animals. Blood pressure, heart/respiratory rates, peripheral oxygen saturation, facial expressions, ocular and extremity movements and presence of crying were also evaluated.	The performance of children with spinal muscular atrophy was below normal. There was attention improvement. Patients had difficulty following the objects and showing other communicative responses. Five patients improved their eye-tracking performance. Stimuli were well tolerated, with no change in baseline variables.	The study did not perform standardized ophthalmologic evaluation. **Bias due to confounding.** The study did not include a healthy control group; the sample was heterogeneous and small. **Bias in selection of participants.** Only qualitative data were available. **Bias in measurement of outcomes.** Some patients were not evaluated or followed. **Bias due to missing data.**
**Authors/Year/Title/Study design/Overall bias**	**Participants**	**Evaluation protocol**	**Main findings**	**Risk of bias**
Polido GJ, Barbosa AF, Morimoto CH, Caromano FA, Favero FM, Zanoteli E, et al., 2017^6^ *Matching pairs difficulty in children with spinal muscular atrophy type I* Cross-sectional **Low risk of bias**	12 children with spinal muscular atrophy type I (3 to 9 years) 12 healthy children (3 to 9 years)	Matching pairs in four tasks of increasing complexity (task 1: matching pairs of same figures; task 2: matching a figure with its corresponding color; task 3: matching letters in uppercase and lowercase; task 4: matching arabic and written numerals). The task involved **attention, spatial location processing and executive function.** An eye-tracking device (Tobii P10) and a notebook were used. Responses were detected by the tracker when children gazed at the stimulus for two seconds.	The performance of children with spinal muscular atrophy was below normal. There were no differences between groups in number of correct answers on task 1 (which had few pairs). On the other three tasks, children with spinal muscular atrophy had poorer performance than controls (less accuracy and longer times). Children with spinal muscular atrophy had more difficulty when the number of stimuli and complexity of the task were increased.	The study did not perform standardized ophthalmologic evaluation. Some children may have performed poorly because they were not familiar with the equipment. **Bias due to confounding.** The study evaluated only one type of spinal muscular atrophy. **Bias in selection of participants.**

Due to the heterogeneity of samples (involving types I, II and/or III of SMA) and testing protocols, we could not run a meta-analysis of results.

## RESULTS

The main findings of the nine studies are presented below and the risk of bias, as classified by ROBINS-I, is shown in Table 1.

Seven studies were cross-sectional protocols,^6^^,^ 7 ^,^ 10 ^-^ 14 one was a cohort study^15^ and there was also a case series.^16^ The studies described the cognitive aspects of children and adolescents with SMA types I, II and III. Overall, three studies described cognitive performance as normal.^10^^-^ 12 In addition, three studies reported that the cognitive performance was above average in some aspects.^7^^,^ 13 ^,^ 14 Three studies described cognitive performance as below average.^6^^,^ 15 ^,^ 16 (Table 1).

## DISCUSSION

The present study evaluated the evidence in the literature about cognitive outcomes in children with SMA. We found that three studies described the cognition of children with SMA as normal,^10^^-^ 12 three as above average,^7^^,^ 13 ^,^ 14 and three as below average.^6^^,^ 15 ^,^ 16 Most of the studies concluding that cognition was normal^10^^-^ 12 or above average^7^^,^ 13 ^,^ 14 evaluated language,^10^^,^ 11 ^,^ 14 visuospatial perception,^6^^,^ 7 ^,^ 10 ^-^ 14 and memory.^8^^,^ 10 ^-^ 14 Children with SMA type I were included in only two of these six studies, and only if they were able to perform hand movements and verbal responses.^7^^,^ 11 Three of these six studies were from the same group and evaluated similar tasks, involving language skills acquisition.^12^^-^ 14

The studies which observed that children with SMA had below average cognitive performance investigated visual and auditory attention^6^^,^ 16 and executive function.^6^^,^ 14 Children with SMA type I were included in all these studies. Therefore, the results of this systematic review suggest that children with SMA type I are more likely to have cognitive impairment (attention and executive function deficits).^6^^,^ 14 ^,^ 16

This difference between SMA types was reported by Sieratzki & Woll (2002).^2^ They wrote a letter to the editor, reinforcing the findings of von Gontard et al. (2002).^7^ They stated that, although cognition was normal, there was a difference between SMA types. Sieratzki and Woll^2^ concluded that the attention of children with SMA type I, who suffer from more severe paralysis, was strongly focused on the approach to one target. Conversely, children with SMA type III had higher functional independence and were able to scan their surroundings for interesting targets.^4^

The following pattern was observed in the review: from 1992 to 2002, studies concluded that cognition was normal,^10^^-^ 12 from 2002 to 2009 studies concluded that cognition was above average.^7^^,^ 13 ^,^ 14 More recent studies reported that cognitive performance was below average.^6^^,^ 15 ^,^ 16 This pattern suggests that previously unreported cognitive impairment in SMA (mainly in type I) is being detected. These findings may be due to the development and application of new cognitive evaluation protocols, which include patients with very limited motor skills.

Samples were highly heterogeneous in terms of age and SMA type (Table 1). Some studies did not include children with SMA type I^10^^-^ 15 (or excluded some children with SMA type I),^11^^,^ 12 because evaluation required hand coordination and/or verbal responses. Only Polido et al.^6^ described the sample education. Only three studies used the same scale (Wechsler Intelligence Scale for Children),^7^^,^ 10 ^,^ 11 and therefore metanalysis was not possible. The other tests used involved several aspects of cognition, such as Language (MacCarthy intelligence scale for children;^10^ North syntactic screening test;^10^^,^ 11 Alouette test for reading age;^10^ Batterie d’evaluation du langage;^10^^,^ 11 Test de Vocabulaire Actif et Passif^11^); Visuospatial skills (a toy was hidden in one of three cups aligned horizontally on a tabletop, which was rotated 180 degrees, and the child had to find the hidden toy;^12^ choice of visuospatial terms to describe object placement^13^); Attention-memory (selective auditory response;^10^ Batterie d’Efficience Mnésique;^10^ North syntactic screening test;^10^^,^ 11 Kaufman assessment battery for children;^7^ following objects trajectories^15^); Reading ability (La pipe et le rat;^11^ reading aloud test);^11^ Executive function (Raven colored and standard progressive matrices;^7^ Functional Independence Measure for children;^15^ matching-pairs task^6^).

Barja et al. (2013)^16^ and Polido et al. (2017)^6^ developed protocols that did not depend on hand coordination and speaking. In these two studies, responses involved eye movements, detected by an eye-tracking device and only children with SMA type I participated. Barja et al.^16^ evaluated and trained attention by showing audiovisual stimuli for four weeks, twice a day, with an eye-tracking device. Although there was attention improvement, they found that patients had difficulty following the objects and showing other communicative responses. Polido et al.^6^ used an eye-tracking device to evaluate matching-pairs skills on four tasks of increasing complexity. The tasks involved attention, spatial location processing and executive function. The authors observed that pair-matching performance was poorer in children with SMA than in controls (less accuracy and longer response times).

Children with SMA type I may need mechanical ventilation, nursing care, gastrostomy, tracheostomy, and therapies (motor and respiratory physiotherapy, occupational therapy, speech therapy for alternative communication and dysphagia). All these adaptations are fundamental for their cognitive-motor development, but do not assure normal/typical development, as the interaction with the environment remains restricted.

As treatment (drugs and rehabilitation) becomes more effective, children with SMA (mainly individuals with types I and II) survive longer.^17^^,^ 18 Facing these new outcomes, health professionals must think beyond survival. Specialized assessment and intervention can help children to interact and develop their cognition.^6^^,^ 16 If cognitive performance is preserved, or even optimized, children with SMA can compensate for the poor motor repertoire and oral communication, achieving greater independence, autonomy and quality of life. The diversity of cognitive assessment protocols made comparison difficult, since each study addressed specific aspects of cognition. Standardized tools are needed to assess cognitive skills. Data on individuals with different types of SMA must be evaluated and analyzed separately. Children with SMA type I should be assessed with eye-tracking devices.

Cognitive performance is directly related with motor skills, for example, locomotion. In the study by Dunaway et al. (2012),^19^ family members were instructed to stimulate children with SMA to use a power wheelchair. Two scales of mobility and quality of life were applied. Five one-year-old children with SMA (four diagnosed with type II and one with type I) participated. Children achieved independence on all items of the mobility scale and had increased scores on the quality of life scale at the age of eight months. The authors concluded that cognitive skills are required to use a power wheelchair. Children with SMA, usually type II, can use power wheelchairs before the age of two, which favors cognitive and motor development.^19^ More specifically, there is a relationship between self-locomotion and spatial language terms,^19^ and language was described as preserved in all studies that included tests for this aspect of cognition.

No studies addressed the influence of genetic mutation characteristics of children and adolescents with SMA on cognitive outcomes. Given we observed that the clinical type of SMA seems to influence cognition, cognitive outcomes might also be correlated with mutation type and/or the number of SMN2 gene copies. Furthermore, although SMA is widely known as a spinal motor neuron disease, recent evidence has suggested it may be considered a multi-organ disease.^3^ SMN1 is expressed in the brain and rare cases of SMA with no SMN2 (SMA type 0) develop white matter and hippocampus atrophy.^3^ These patients also show thalamus and basal ganglia hyperintensity on resonance magnetic imaging.^3^ Therefore, the severe reduction in SMN protein levels might lead to progressive encephalic dysfunction and degeneration.^3^

This systematic review showed that there is no evidence of cognitive impairment in children with SMA types II and III. Although there is little evidence in the literature on the cognitive performance of children with SMA, children with SMA type I are more likely to be affected. Our findings showed that attention and executive function seem to have higher risk of being impaired. More tests must be developed for properly assessing the cognition of patients with SMA, especially for more severe forms. Cognition can be affected in children who face environmental restrictions,^20^ due to severe weakness and poor oral communication. Attention and executive function assessments and follow-ups are fundamental to assure healthy cognitive development.
